# Making primary healthcare delivery robust for low resource settings: Learning from Mohalla Clinics

**DOI:** 10.1007/s44155-022-00030-0

**Published:** 2023-01-10

**Authors:** Md Haseen Akhtar, Janakarajan Ramkumar

**Affiliations:** Department of Design, Indian Institute of Technology, Kanpur, India

**Keywords:** Mohalla clinic, Primary health care, Healthcare delivery model, Remote population, Mobile primary health center

## Abstract

The present healthcare scenario is still in its compromised state, whether it is the lack of infrastructure, medicines and human resources, especially in rural India. Moreover, the condition worsens in rural areas due to several reasons like lack of awareness, proper roads to access, and lack of proper delivery of healthcare model. The state government of Delhi, India, set up the “Mohalla” Clinics to provide essential healthcare to residents of Delhi and the surrounding areas, focusing on the urban poor. Essential health services, such as vaccinations, family planning, and counselling, are available at the Mohalla Clinics, where a doctor, a nurse, a pharmacist, and a lab technician are staffed. Despite a strong start and low operating costs, the Mohalla Clinic initiative still struggles to cover all Delhi state as envisioned. This study analyses the operational challenges of Delhi’s “Mohalla” Clinics and proposes lessons to be implemented for other primary healthcare infrastructure services for remote areas. The analysis is based on the systems (infrastructure, facilities, and services) strengths and limitations from a literature review and qualitative interview conducted among 55 respondents, including doctors, nurses, and patients among 11 Mohalla Clinics using the SUTD-MIT (Singapore University of Technology and Design—Massachusetts Institute of Technology Industrial Design Centre) interview template for Product Service System (PSS). The results show that there are lessons to learn from the model of Delhi Mohalla Clinics for other states to implement in their primary healthcare sectors. To achieve Universal Health Coverage (UHC), the Delhi Mohalla Clinic falls short due to several limitations. Thus, to achieve UHC, the Indian healthcare system needs a new healthcare delivery model. Hence, we ought to propose a new healthcare delivery model based on the gained insights from the study. One such delivery model proposed is a mobile Primary Health Center (mPHC). This collapsible system can be taken to far-flung regions, deployed for some hours, run the Out-Patient Department (OPD), collapsed, and returned to base.

## Introduction

It has been a decade since poliomyelitis, yaws, and maternal and neonatal tetanus were eradicated from India’s healthcare system [1]. There is still a high presence of communicable diseases like tuberculosis, measles, emerging and re-emerging diseases, the increasing number of noncommunicable diseases like diabetes and hypertension, and human resource deficiency for health, among other things [1]. Provider availability, guaranteed services, drugs, and diagnostics are absent and referral links are inefficient in India’s healthcare system. A considerable number of people seek treatment for minor ailments such as fever, cough, and cold at government health institutions are still not satisfied. Dissatisfaction with public health services has resulted from overpopulation, long waits, and several other reasons. Seek care from unqualified or private physicians, even if it costs them money, is how people, including the lowest fifth of the population, feel about public health institutions [2, 3]. Through government funding, India’s National Health Mission (NHM) has helped increase attention to health care marginally over the previous 10–15 years, but there is still a long way to go. A new healthcare delivery model was proposed by the Delhi government to set up clinics which has gained national and international interest in the form of Mohalla Clinics [3, 4]. Different political parties of several Indian states have expressed a desire to construct such Mohalla Clinics [5, 6]. The healthcare system in Delhi, India, suffers from a lack of regular access to healthcare professionals, medications, and diagnostics, as well as from a lack of referrals to more advanced facilities [7]. When the primary healthcare system is underfunded and overstretched, many people end up going to secondary and tertiary care institutions for minor ailments [2]. The well-established private sector in Delhi also attracts a significant number of people [8]. As a result, overcrowding, long wait times, subpar care, and high out-of-pocket expenses (OOP) are all issues of concern for the underprivileged to afford key healthcare services.

To help India achieve Universal Health Coverage (UHC) and develop its health systems, Mohalla Clinics may be a useful tool. A partial success, these clinics have brought healthcare to the forefront of political debate, but it is still a long way from where it was with the Bijli Sadak Pani (BSP) of nearly 15 years ago (or electricity road water). In India, as more states implement health-related policies, it is probable that BSP would be replaced with Swachchata-Swashthya-Shiksha-Safaai-Saamaajick-shetra initiatives.

## Health system in Delhi

**Fig. 1:**
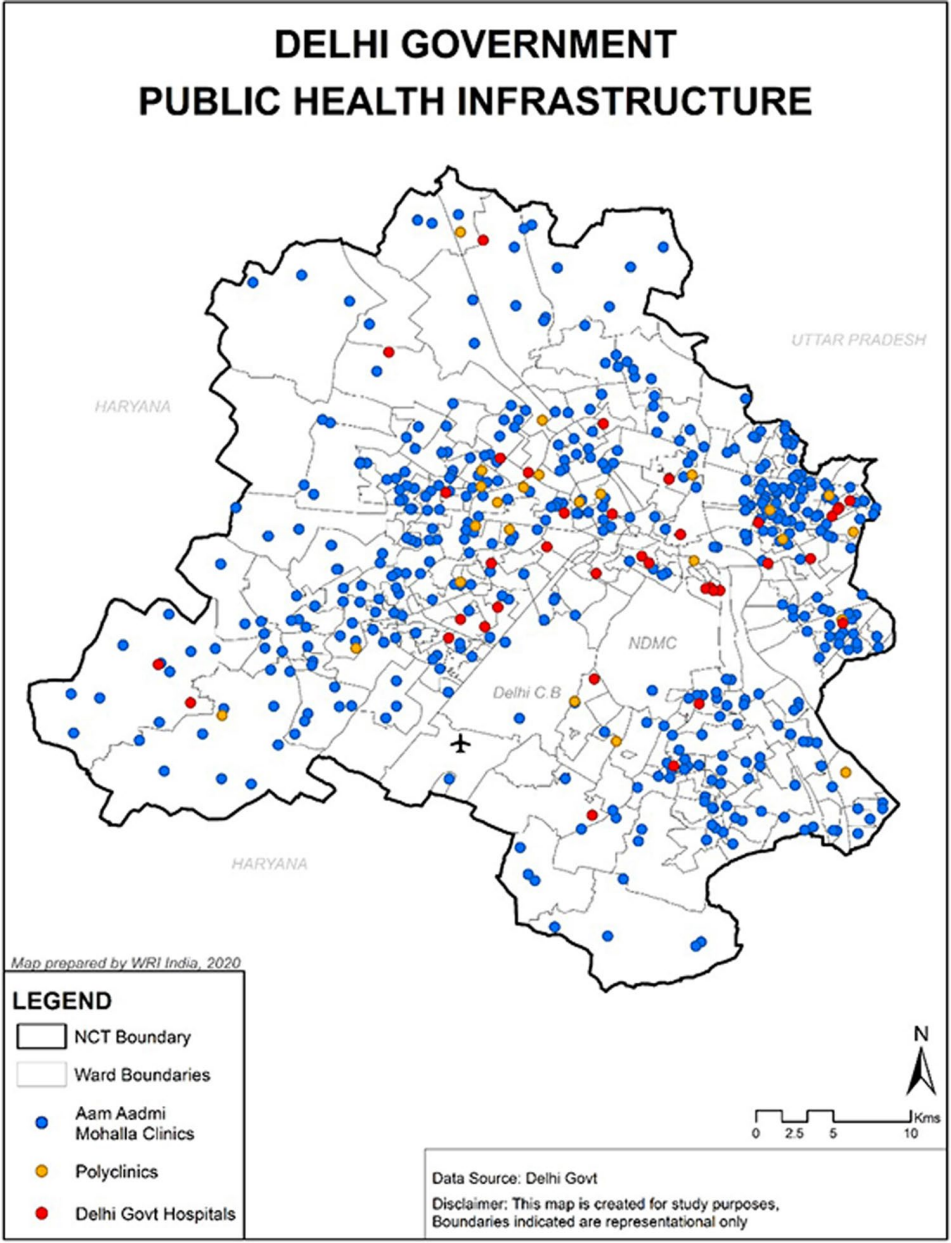
Delhi government public health infrastructure (proposed by W.R.I. India, 2020)

Delhi is India’s capital city, with a population of 16.8 million, 97.5 percent of whom live in urban areas, a land area of 1483 square kilometer, and a population density of 11,297 (varying from 3800 to 37,400/square kilometer). Over one-tenth of the city’s population (1.8 million people) [9] lives in slums, the majority of whom are new arrivals from other regions of India. Delhi is the world’s third-largest metropolitan region in terms of population. More than a dozen organizations provide health care in Delhi, which includes the three municipal corporations. The number of healthcare facilities in Delhi (as shown in Fig. 1) varies as there were 88 general hospitals, 1298 dispensaries, 230 maternity homes and subcenters, 54 polyclinics, 1160 nursing homes, and 124 specialized clinics as of March 31st, 2017[10]. In addition, fifteen allopathic medical schools are run by the government. Nearly 12,000 hospital beds, over 200 dispensaries, and several polyclinics are all owned by the Delhi government, which accounts for approximately one-fifth of the city’s health facilities. Approximately 33.5 million outpatients and 0.6 million (600,000) inpatient patients are examined and treated by government-run health institutions in Delhi each year. One can find a huge number of private hospitals and clinics around the city-state. Per capita government health expenditure in Delhi state in 2012–13 was 1753 INR, while the average for major Indian states was 737 INR. The government is responsible for a 68 percent of the remaining health care costs [11]. Hospital treatment in metropolitan areas is provided by the private sector at a rate of approximately 55 percent. Private outpatient treatment was provided to 87 percent of males and 71 percent of females in Delhi (national average 76 percent and 73 percent, respectively) [12].

**Fig. 2:**
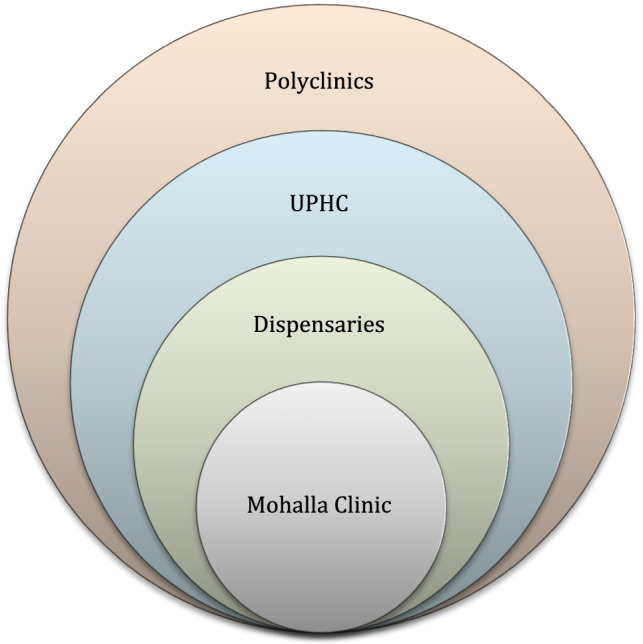
A conceptual hierarchy model of existing healthcare delivery services in Delhi

At least in their current form, Mohalla Clinics are widely used as a euphemism for primary health care. Primary health care encompasses everything from health promotion and disease prevention through treatment and rehabilitation, as well as public health services. To be more specific, public health focuses on disease prevention, whereas clinical services focus on disease treatment. Improved sanitation is a top priority for some places, while nutrition and healthy lifestyle education are high priorities in others. The current architecture of Mohalla Clinics plays little emphasis on public health services or the needs of the general people. There is a lack of attention paid to basic needs such as cleanliness, safe drinking water, personal hygiene, and nutrition education in these clinics. Curative services are the focus of Mohalla Clinics, which cannot be considered comprehensive primary health care in its current form. While the Mohalla Clinics focus on curative and diagnostic services, Urban Primary Health Centers (UPHCs) under the National Urban Health Mission (NUHM) [32] could provide public health services with well-established referral links in addition to curative treatments. It’s also worth considering how UPHC and Polyclinics work together. More than half a million individuals could be served by the UPHC, and polyclinics coupled with three-four lower-level facilities like Mohalla Clinics. Convergence between state-owned programs and Ministry of Health and Family Welfare (MoHFW) efforts is a sustainable option for healthcare in countries like India, where health is state responsibility, and the central government advises on policymaking and implementation. Mohalla Clinics, Dispensaries, UPHCs, and Polyclinics all work together to strengthen primary healthcare systems in the state of Delhi (Fig. 2).

## Delhi Mohalla Clinic—History, Vision, Design, Planning and Implementation, Budget and Cost

### Mohalla Clinic—History

When the new state administration in Delhi entered office in February 2014, it was expected that basic healthcare would be provided to the city’s urban population through “Mohalla” Clinics (as they are called in the local language) [7, 8]. A physician, a nurse, a pharmacist, and a laboratory technician work in these clinics to provide outpatient consultations, free medicines, diagnostics, vaccinations, family planning counseling, and other services to low-income individuals [7, 13]. With a specific focus on the urban poor, the idea was to provide high-quality, affordable healthcare in the patient’s community. With the lofty goal of creating 1000 clinics in diverse Delhi assembly constituencies, the first clinics were launched in July 2015 [4]. The decision was revealed in the state budget for 2015–16 to establish 500 clinics in the first year. The inaugural Mohalla Clinic was established on July 19, 2015, in the Peeragarhi neighbourhood of northwestern Delhi. The government has pledged to construct between 500 and 1000 clinics (or 14 clinics per assembly constituency). The Peeragarhi Mohalla Clinic is located within a cluster of jhuggi jhopri (slums). The clinic reportedly cost the government twenty million Indian rupees [7].

The ‘Mohalla’ Clinics established by the Delhi government allowed residents, particularly the urban poor, to receive healthcare closer to home, reducing travel time and waiting time. More than 110 vital drugs and over 212 diagnostic tests were made available at no cost to those who could not afford them [14]. The clinics eased the financial burden on low-income households by providing free healthcare. Because of Delhi’s high population density, the clinics could operate at a lower cost per patient than a tertiary hospital would have. Each clinic cost roughly 31,000 US dollars to start [7]. For each patient they treated, they were paid 0.4 US Dollars. The clinic’s success was further aided by the abundance of doctors and nurses in Delhi, a major metropolis. Counseling and referrals were also available at the Mohalla Clinic. As a result of these clinics, Delhi’s secondary and tertiary healthcare institutes experienced less patient congestion [13, 15].

### Mohalla Clinic—Vision

A community clinic in a slum neighborhood in Delhi was the starting point for the Mohalla program [7, 16, 17]. It was inspired by the widespread use of Mobile Vans (MVs) and Mobile Medical Units (MMUs) that we came up with this notion. Finally, it was bolstered by the top level of government’s intention to execute their promises and obligations to develop healthcare systems rather than delivering ad-hoc solutions in the form of Mobile Medical Units (MMUs) and Mobile Vans (MVs).

Mobile Medical Units (MMUs) and Mobile Vans (MVs) were part of the inspiration for Mohalla Clinics. In addition to India, these MMUs can also be found in other nations like South Africa [18], Greece [19], Zambia [20], and Saudi Arabia [21]. Medical supplies, doctors, and other staff are transported to patients in underserved areas via a fleet of modified or adapted tempo vans and other vehicle types. Delhi administration decided to rapidly extend the state’s network of MMUs by introducing a few additional MMUs. These MMUs and the regions where they would provide services, such as underserved areas, unlawful colonies, and clusters of migrant population colonies, are typically funded by the state governments with help from the union Ministry of Health & Family Welfare (MoHFW). These van-based clinics received an incredible reaction from the communities they served, and there was a tremendous demand for services. MMUs delivery of health services is not only unpredictable and dependent on a range of external circumstances, such as the availability of vehicles, doctors and road conditions at that time government officials understood that it may also be unsustainable in the long run. Administrative and procedural challenges associated with purchasing several vehicles and recruiting contract staff, including physicians, were also regarded to be limiting concerns. An appropriate and a more long-term solution was needed, one in which services were based in the community and people knew where to go for services, as well as assurances of the availability of providers, medicines, and a comprehensive service package with adequate community linkage were all components. MVs and MMUs were at best viewed as stopgap measures, and they couldn't accomplish much of this. Therefore, the authorities after several discussions, backed the Mohalla Clinics idea.

First Mohalla Clinic in Delhi opened in Peeragarhi, West Delhi, on July 19, 2015. The establishment of an extra 100 clinics took an additional 9 months. As of December 2016, 55 of the state’s 70 assembly districts and all eleven districts had been served by 106 clinics [16]. There had been plans to build a thousand of these clinics in Delhi by the government. Despite considerable political support, just 10 percent of the planned number of these clinics were constructed by the December 2016 [22, 23]. The establishment of the anticipated number of clinics has been delayed due to several factors, including inadequate planning ahead of time (no operational plan was created until 1 year into implementation), difficulty choosing the locations (the land is not under the state government’s control), delays in approvals at various levels, and frequent changes in technical leadership within the health department.

First, a Portacabin structure was built on government land, but the plan to acquire land ran into difficulties, and approximately 100 clinics were erected in rented or rent-free private dwellings. Attempts to speed up the process by opening these clinics in government schools ran into administrative roadblocks and could not be implemented until the end of 2016, requiring authorization from officials. Most clinics opened in 2016 and soon became popular in the community [24]. According to government, approximately 800,000 people have received health care in Delhi by July 2016 and 43,000 pathological tests had been completed in the prior 5 months [25]. On average, each clinic saw 70 to 100 patients every day. As a result of the dengue and chikungunya outbreaks that occurred in Delhi in September and October of 2016, where health facilities were overrun with patients, Mohalla Clinics became an important entrance point for patients seeking medical attention and undergoing dengue lab testing. The city’s situation was lessened because of this major alleviation for huge healthcare facilities [26]. Some 1.5 million people visited these facilities at the end of 2016, and most had been open less than a year [13]. External experts, opposition groups, and media all came to investigate the clinics, which drew attention to themselves. Most of these people complimented the concept and mentioned the great demand for the services provided by these clinics as a reason for their positive comments. The Lancet observed in an editorial published in December 2016 that a network of local Mohalla Clinics are successfully serving individuals that would otherwise lack access to health services [27]. According to a wide range of publications, these clinics are in line with the ideas of Universal Health Coverage (UHC), and they help the poorest people get better healthcare and lessen their financial burdens [7, 28]. According to Delhi government, 40–50 percent of the patients at these clinics were first-time visitors to a government health institution. The lower patient load at Mohalla Clinics has been noticed by many clinics run by unqualified practitioners. Almost universally, it is agreed that these clinics have helped the poorest of the poor get better access to health care from trained professionals, although this must be examined and documented more thoroughly [13].

### Mohalla Clinic—Design

**Fig. 3:**
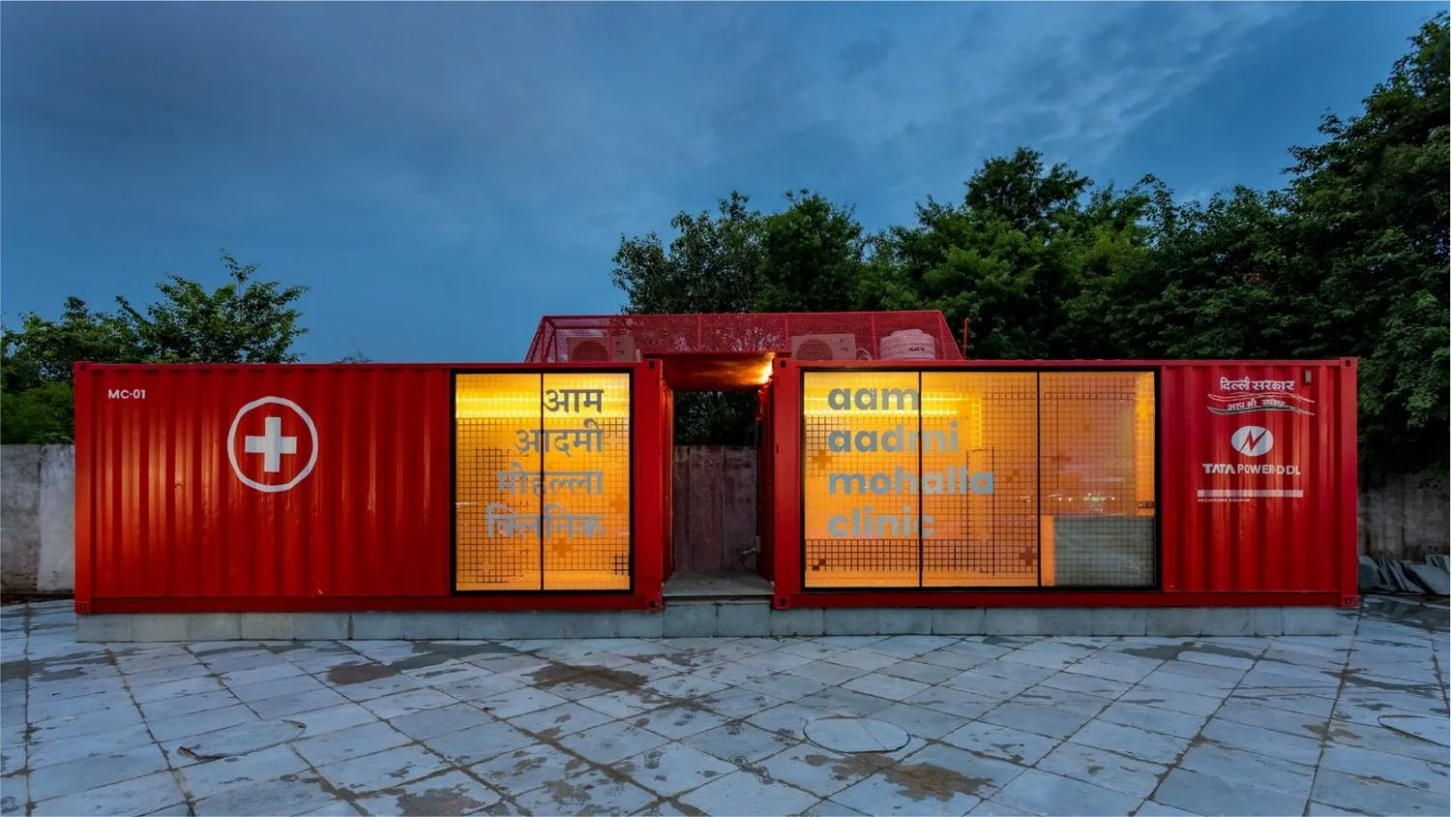
Shipping container Mohalla Clinic: Box type

These Mohalla Clinic units include outpatient consultations, free medications and diagnostics, immunizations, family planning, referral, and counseling services. In the future, weekly visits by specialists such as gynecologists and ophthalmologists are planned. On an area of roughly 50 square yards, a two-room clinic constructed of prefabricated materials (portacabin) has been erected. It has access for an ambulance to approach, as well as green area surrounding the facility. The clinic is equipped with a doctor’s office, a pharmacy, laboratory testing kits, and a token vending machine. There is a cable-connected television, a water dispenser, and a completely air-conditioned waiting area with chairs [7]. There are three types of infrastructure for a Mohalla Clinic in Delhi. These are portacabin in 3 different volume types (Hut type, Box type and L-shaped type), shipping container type (Fig. 3), and space on lease. Depending upon the space constraints, different modules has been used. The Portacabins hut types are deployed in areas where there are unused pedestrian parks, box type is deployed near bus stops and L-shaped are deployed in open grounds near public schools, colleges, and post offices. The shipping container types are new types deployed near open public spaces such as squares, plazas, etc. with the advantage of portability and same type of services in half of the space (350 sq. ft. against 600 sq. ft. of portacabins).

To have a different perspective into the system, we also looked at literatures so that we can compare our findings with the reported results about Mohalla Clinic. The notion of Mohalla Clinic offers several well-known advantages and downsides that make it a viable health intervention (Table 1).

**Table 1:** Comparative analysis: Mohalla Clinic and existing infrastructure

Existing health facilities	Mohalla clinic
1. Health positions: limited resources, reliant on financing, Limited range of guaranteed services, understaffed, underutilized by community members, not always in regions of deprivation and underservice facilities with a specific focus, such as mother and child welfare centers. focused on small and distant target populations in cities like Delhi 2. Mobile Vans or Mobile Medical Units (MMUs) would travel to a specified location and offer services, usually to underserved populations, however the nature of the offerings varies 3. Dispensaries and polyclinics: these are frequently located in buildings with numerous rooms and are either overcrowded or underutilized, or just a small number of patients who use them can be certain of the availability of services and providers	1. Supported by state government’s dedicated funding, the vast network, and the guarantee of a variety of services and suppliers 2. Located in underserved areas and slums 3. A variety of personal healthcare services where any family member can access essential medical care 4. Fixed physical infrastructure, so people know where to go to receive healthcare 5. These clinics would consist of two to three rooms and offer services. Efficiency in service delivery

A few Indian governments (such as Maharashtra and Gujarat), as well as a few municipal corporations (like Pune) have expressed an interest in developing some form of these clinics in their respective states [5, 6, 29–31]. There are at least two “proofs of concept” proving the clinics’ effectiveness. There is a tremendous demand for these clinics’ services from individuals who have “voted with their feet.” As a second piece of evidence, various Indian states have expressed an interest and willingness to build health facilities with a similar design. Health systems view these clinics favorably from the perspectives of, for example: accessibility; equity; quality; responsiveness; and financial security, further explained as below:

- Accessibility: increase access to high-quality health services for all
- Equality: focus on poor and marginalized segments of society who are disadvantaged when receiving health care
- Ensuring that services meet people’s expectations and are delivered in accordance with standard guidelines.
- Comprehensive assurance of essential health services: moving beyond a limited package of services to guarantee coverage for additional diseases and conditions.
- Financial protection: reduces all costs to the people and makes health care affordable, so that people do not become impoverished because of accessing these services. No upfront direct or indirect payment for health services.
- Community participation: active engagement of community members in selecting and identifying sites for these clinics, fostering the necessary sense of ownership.

A variety of government policy initiatives to reform healthcare service delivery were introduced at the same time as the Mohalla Clinics, but they got less attention. Some of them were establishment of Delhi healthcare companies, program of free medicines and diagnostics, elimination of private beds in hospital settings. Redesigning a three-tier system of service delivery and referral into a four-tier system and start of Mohalla Polyclinics were some of the initiatives on the infrastructure development level. Centralized accident trauma service ambulances, free trauma care services, private sector physicians on call for government health facilities, no administrative positions for doctors, allowing them sufficient time for patient care, and technical or subject matter experts as departmental heads were other initiatives on the human resource part. Effective utilization of resources, expanding access to health services, elimination of user fees at government hospitals, lowering the out-of-pocket costs for individuals, ban on all forms of gutkha and tobacco products, and making the services more affordable were all directly impacted by the government’s decisions. Due attention has also been given to the design of these clinics to make them more user-friendly with design evolution over time (Table 2).

**Table 2:** Design evolution of Mohalla Clinics

Started as	Changes overtime
1. Portacabins that are prefabricated are used for housing 2. Originally designed as two rooms with open seating space for patients 3. All clinics employing government physicians 4. Involvement of private doctors on a fee-for-service basis is also permitted 5. Select weekly visits to specialists such as gynecologists and ophthalmologists for basic services 6. Opens for 4 h in the morning	1. Considering the local conditions (summer heat and security concerns), waiting areas were made closed and a third, if possible air-conditioned, room is added or constructed 2. Availability of restrooms in the waiting area. In rented or free locations 3. In addition to preventative and curative services, it became a designated facility/fever clinic for dengue during the outbreak 4. Minimum 4–6 h, with flexible scheduling and evening shifts as available

### Mohalla Clinic—Planning and Implementation

These Mohalla Clinics are designed and deployed with several intentions and goal mentioned as follows:

- Accessibility: healthcare should be free and accessible to all residents within a few minutes’ walk of a health center that is open for at least 6 h a day and has access to basic health services, medications, and diagnostic testing. In this way, the number of patients, who require referrals can be reduced by as much as 89 percent. At least 10,000 to 15,000 people will be served by each clinic in the Jhuggi Jhopri community.
- Minimum staff required: there must be at least one certified doctor, one auxiliary nurse midwife, and one pharmacist present in a Mohalla Clinic.
- Providing services: basic first aid, mother, and child health care services, including immunization, prenatal and postnatal care, family planning counseling, and referrals to the next level of specialized treatment facilities, are included in a comprehensive healthcare package. They also have plans to establish national health programs through these clinics, as well.
- Referrals to specialists and continuity of care: weekly availability of specialists (pediatrician, gynecologist, and ophthalmology) is recommended. It has been suggested that a tiered system of referrals to health facilities be implemented (though yet to be made fully functional)
- Diagnostics and pharmaceuticals: 108 drugs and more than 200 diagnostic tests from an approved list are available to people who need them, free of charge. These underserved areas include slums and the Jhuggi Jhopri colony, which have been designated as slums, housing for migrants and the impoverished. At 400 m from the main road in the center of Jhuggi Jhopri hamlet in northwestern Delhi, a first such clinic was created. The locations are determined by the local community, resident welfare associations, surveys conducted by the planning department, and site verification by a group of health professionals.
- Physical infrastructure and ease of access: the utilization of two or three rooms has been proposed. Private homes with comparable amenities or prefabricated portacabins could house the rooms. At least one of the rooms will be designated for use by a doctor, who will have access to a private examination room. As a laboratory, pharmacy, and waiting room, the second room is used by patients while they are waiting for their turn to see the doctor in the first room. Waiting areas could be set up in a third room if it is available, but if not, the roofed open space should be used. With these facilities, providing a drinking water dispenser and a restroom, the design must incorporate features like central air conditioning and a television with a cable connection. These clinics, which must be situated on an all-weather, ambulance-accessible road in a wide-open area, and must be easily reachable by patients. The construction of each clinic was estimated to cost around 30,000 US dollars. However, most clinics used rented space until December 2016. These clinics’ operational costs have not been analyzed, according to reports.
- Use of computer technology: patient queues are managed by a token vending machine (like those found in bank lobbies), records are kept on computers for each person in the practice, prescriptions are written on tablets or software programs using tablets/software programs, and several laboratory tests are performed using technology-based tablets.
- In charge of administration and management: Delhi Healthcare Corporation, the state’s health bureaucracy’s top official, oversees implementing this government initiative, which was spearheaded by the state’s minister of health and other high-ranking officials.
- Public–private partnership: these clinics are run by private doctors who are paid on a “fee for service” basis. Patients pay a consultation charge of 30 INR. An additional 10 INR is charged for each patient, if an assistant is present during the procedure. As an outpatient clinic, these doctors are given access to a ready-to-use chamber that can be used in a matter of hours.
- Working hours and schedules: 6 h maximum, with a minimum of 4 h in the clinic. Clinics typically open in the morning, although hours can be changed to meet patient demand, and some even open later at night. Except for public holidays, the clinic is open 6 days a week.
- Financial security via free services: decreased costs due to increased attention and investment in healthcare, ambulance, and transport services; and a strong referral linkage with emphasis on continuity of care are other features.

### Mohalla Clinic—Budget and Cost

A total of 30 million US dollars was set aside for the construction of 1000 clinics. The overall health budget of the Delhi government in 2016–17 on Mohalla Clinic was 784 million US dollars [2]. These clinics must be operational and financially sustainable, but accurate estimates of both the initial and ongoing costs involved with this are needed. The initial investment has been calculated, but the ongoing expenditures of human resources, diagnostics and drugs, and operational expenses, which would add an extra 45 million US dollars every year, have not been included. Users must enjoy a positive experience along the continuum of care for the project to be effective and successful. It was stated that Delhi would have a four-tiered healthcare system with referral links (first, Mohalla Clinics, second, polyclinics, third, multispecialty hospitals, and fourth, super specialty hospitals and medical institutes). The Mohalla Clinics must be considered on par with or even more so than other levels of care. The “continuum of care” is essential, but it is insufficient to retain people in public health facilities; they also need to get high-quality, dependable care. Healthcare services are provided by around 25 different types of health facilities in the state of Delhi. Even program managers have difficulty telling one facility from another when it comes to service delivery and most of these buildings, which are virtually identical [9]. When viewed through the eyes of an outsider with inadequate knowledge, some people may mistake Mohalla Clinics for any other type of healthcare facility. However, the Mohalla Clinics’ well-considered design distinguishes them from conventional healthcare facilities (Table 3). The coordination and harmonization of various types of health facilities run by various organizations is a proactive issue that must be addressed because varied health facilities make access to health services complicated, time consuming, and onerous for the general people. Curative, clinical, and personal health services are the primary emphasis of most currently operating health facilities, leaving them underequipped to address the needs of the public or the general population. Proposals for 150 polyclinics have been submitted as of December 2016, and 23 had already been built; Because of this, it is imperative to have a well-respected and functional referral linkage at all levels.

**Table 3:** Mohalla Clinics concept: Strengths and Limitations

Strengths	Limitations
1. High political buy-in: the state government appears committed to providing citizens with quality health services. These clinics were announced as a flagship program alongside the education sector, possibly a first for an elected government of an Indian state to prioritize health in this manner. 2. The fiscal strength of the state and the relationship between financial planning and allocation benefit Delhi. This initiative appears to have a connection between planning and intent. 3. The state has the financial ability to recruit additional physicians (one each) for these clinics, which may not be possible in other settings despite the states’ desire to recruit and allocate funds (due to shortage of doctors). 4. Equity in service delivery: clinics target underserved populations and regions with limited access to medical care. 5. Large network of secondary and tertiary care facilities: to accept referrals from lower-tier health facilities, referral under these clinics has been proposed but is not yet fully operational. 6. Availability of trained human resources: Delhi has a greater number of doctors and other staff per 1000 people, so the challenges in recruiting staff would be financial and administrative rather than a lack of trained human resources. 7. The need for health services is greatest among the poor and migrant community, which constitutes a larger target population. 8. Token vending machines: medication vending machines demonstrate the responsiveness of the health care system.	1. These clinics, at least until now, have focused on personal or curative, diagnostic, and a small number of preventative health services. 2. Limited connection with community and outreach services: community-based preventive and promotive health interventions should be included in the services provided. 3. Limited linkage with existing mechanisms for service delivery: the state of Delhi has multiple agencies delivering basic health services, and it will be difficult for Mohalla Clinics to be effective unless there is improved coordination among these agencies.

The budget for the fiscal year 2022–23 includes funding for the expansion of Mohalla Clinics and polyclinics, the two pillars of basic healthcare in the nation’s capital. There are already 520 Aam Aadmi Mohalla Clinics and 29 polyclinics in the city, and the government intends to construct 1000 Mohalla Clinics. 5.49 billion patients have been treated at Mohalla Clinics to date. The government recently launched 20 school health clinics on a pilot basis, each of which would undertake a comprehensive health screening monthly. These specialized clinics also employ psychiatrists to evaluate the mental development of the children. The health sector has been allocated 97,690 million INR (13 percent of the overall allocation) for 2022–23, which is 1 percent less than the amount for 2021–22. This amount has been set aside for the construction of new government hospitals and the renovation of existing facilities. After the construction of the new hospitals and completion of the renovations, the capacity will expand by 16,000 beds. A budget of 500 million INR has been suggested for the Delhi Arogya Kosh program, which provides free treatment, surgery, radiology, and diagnostic services at designated private hospitals if comparable services are unavailable in government-run hospitals. In addition to these infrastructure improvements, the government will build a hospital information management system and issue all citizens with health cards. A budget of 160 billion INR has been suggested for these programs. The government also intends to establish a 24-h, toll-free hotline for health card holders that will provide advice on matters such as where to receive treatment for any disease. On the helpline, individuals can also make appointments with hospital physicians. A special budget allocation of 150 million INR has been created to give free yoga and meditation services to the public.

## Study design

**Fig. 4:**
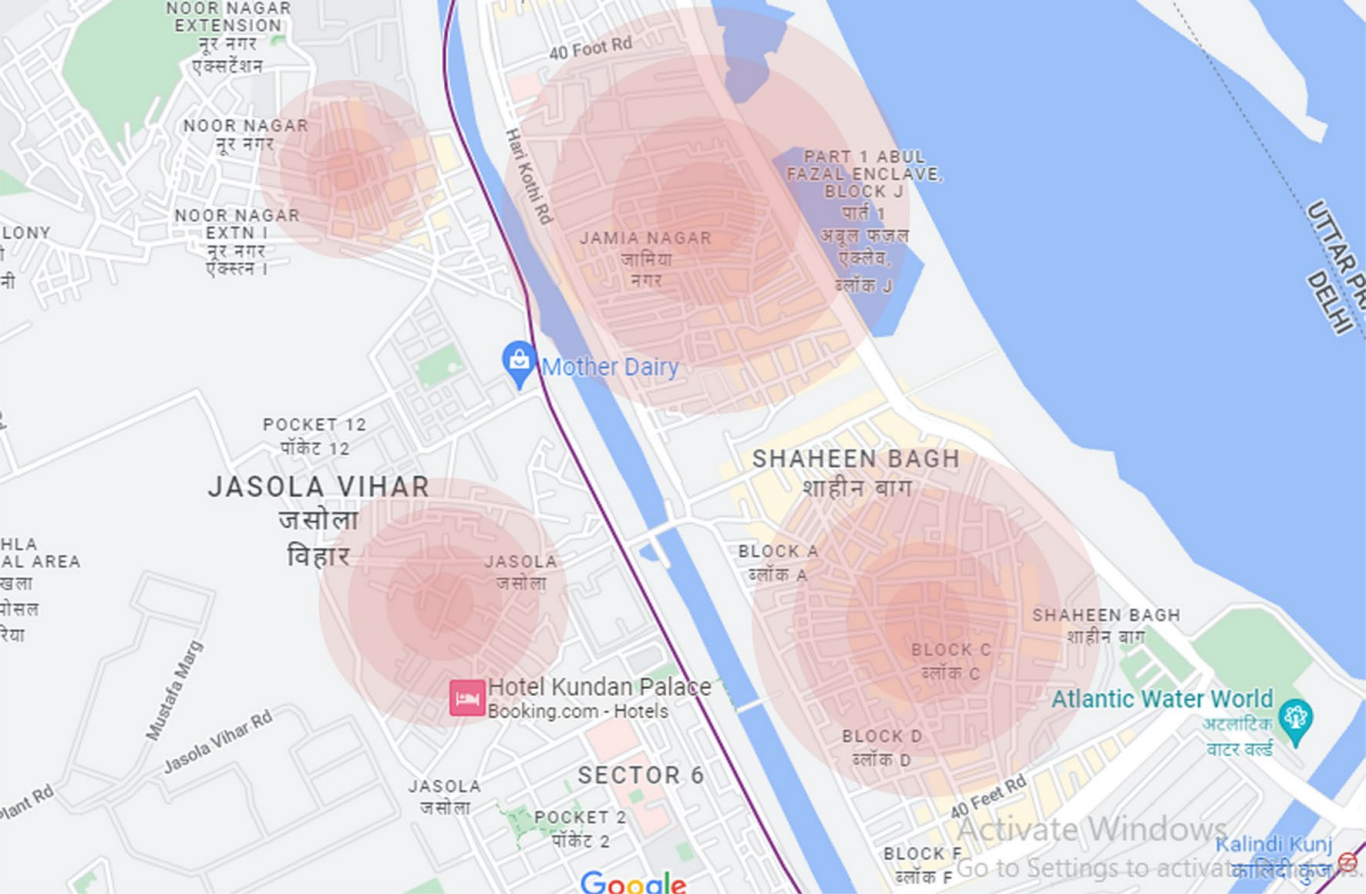
Mohalla Clinics study zones

Eleven Mohalla Clinics in four zones marked on the map were purposively sampled. Each of these Mohalla Clinics were within the radius of 5 km illustrated in four zones as Noor Nagar, Jamia Nagar, Jasola Vihar, Shaheen Bagh (Fig. 4). Two clinics in Noor Nagar, two clinics in Jasola Vihar, three clinics in Shaheen Bagh, and four clinics in Jamia Nagar were sampled. At each Mohalla Clinic, a doctor, a nurse, and three patients were interviewed, making a total sample size of 55 participants.

**Fig. 5:**
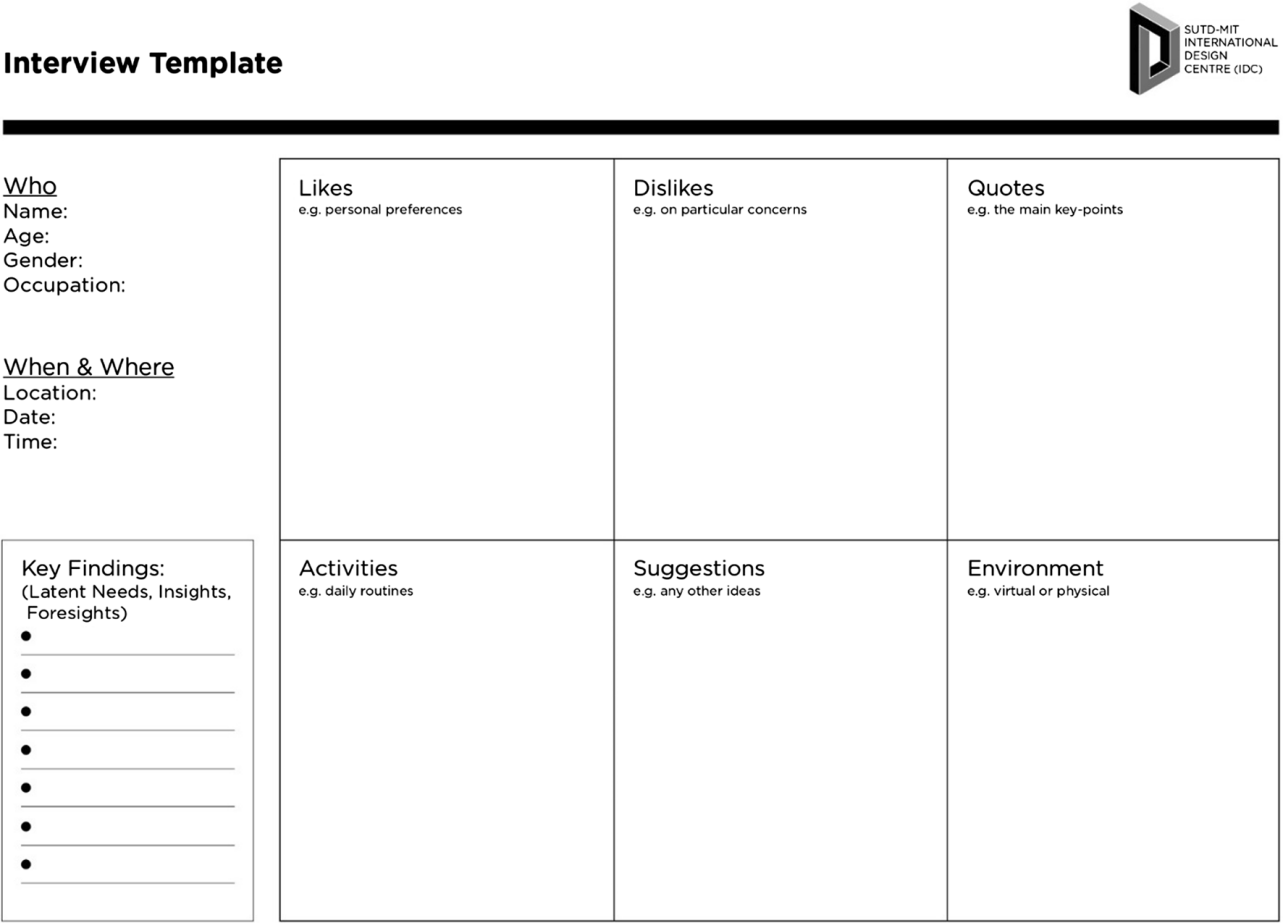
SUTD-MIT interview template for Product-Service-System (PSS)

A qualitative interview was taken enquiring about the infrastructure, facilities and services provided. The SUTD-MIT interview template (Fig. 5) were used for the Product-Service-System under consideration. Author 1 (corresponding Author) conducted the interview. Participants were identified by passive observation and asked for willingness to answer some questions. The interviewer (Author 1) introduced himself to each participant while asking the willingness. It was also ensured that the interviewer informs participants that the data collected will be reported for other to read and use it publicly. Verbal consent was taken from the participants. After the informed verbal consent confirmation from the participants, the first question asked was about the services provided, staff details, and the available facilities (from doctors and nurses). Then the next question is to narrate a typical day at the PHC with all the different activities performed. In the case of patients, it was asked to describe a usual routine when visiting the Mohalla Clinic. The next important question is: What do you like about the overall system at this PHC with a follow-up of dislikes for the same? Lastly, the participants are asked to suggest improving the present situation (if any). If there are quotes mentioned by the participants in between the interview at any point is noted. Author self-observation is also indicated in terms of the overall cleanliness and hygiene of the place. Standardization was ensured by using the same template (SUTD-MIT interview template for PSS) to interview each group of participants. It was ensured that the data were cross-checked before, while and after interpretation by the co-author (Author 2). Interviews were conducted inside the Mohalla Clinic with doctors and nurses while it was conducted outside the Mohalla Clinic with the patients after they had consulted the doctor. The interview was conducted in English (and sometimes Hindi) with doctors and nurses, while it was completed in Hindi with patients. The responses were recorded in English in the template.

## Results

Eleven doctors, eleven nurses, and thirty-three patients were interviewed using the SUTD-MIT template. All of them were asked about the daily activities performed in the Mohalla Clinic, their likes, dislikes, and some suggestions regarding the improvements needed. We received a wide range of answers due to three distinct user groups. Regarding the facilities, each Mohalla Clinic has a waiting area for an average of 20 people capacity, a doctor’s room, a reception, a pharmacy, and a toilet. Some of them (9 out of 11) have a storeroom. The working hours are from 8 am to 2 pm every day. Doctors were hired on a volunteer basis with minimum qualifications of Bachelor of Science and Bachelor of Medicine (MBBS). Experienced doctors are preferred. As soon as the patient arrives, they go towards the reception, where the receptionist takes a photo of the patient from a tablet. The information gets updated for the doctor, who has a similar tablet with the same mobile application specially designed by the government for Mohalla Clinic to maintain the patient’s database. Whatever consultation happens is recorded for a particular patient on every visit. This helps in saving an average of 10–15 min of doctor’s time, which allows him to consult 25 percent more patients in a day. The detailed analysis of the responses from the interview from all three user groups are explained further.

Two nurses and six patient showed dissatisfaction on the Likert scale of 1–5 (Likert scale: 1–5 where 1-highly dissatisfied, 2-dissatisfied, 3-average satisfied, 4-satisfied and 5-highly satisfied). 19 out of 33 patients (57.5 percent) were concerned about travelling to the far Mohalla Clinic due to their preference over the doctors they want to check up with. 3 out of 11 nurses (27 percent) showed dissatisfaction with the location of Mohalla Clinics which are in the Mohalla’s (inner part of societies); they find it difficult to travel during the morning as well as late afternoon time from their homes. This can be a personal workforce dissatisfaction issue.

**Fig. 6:**
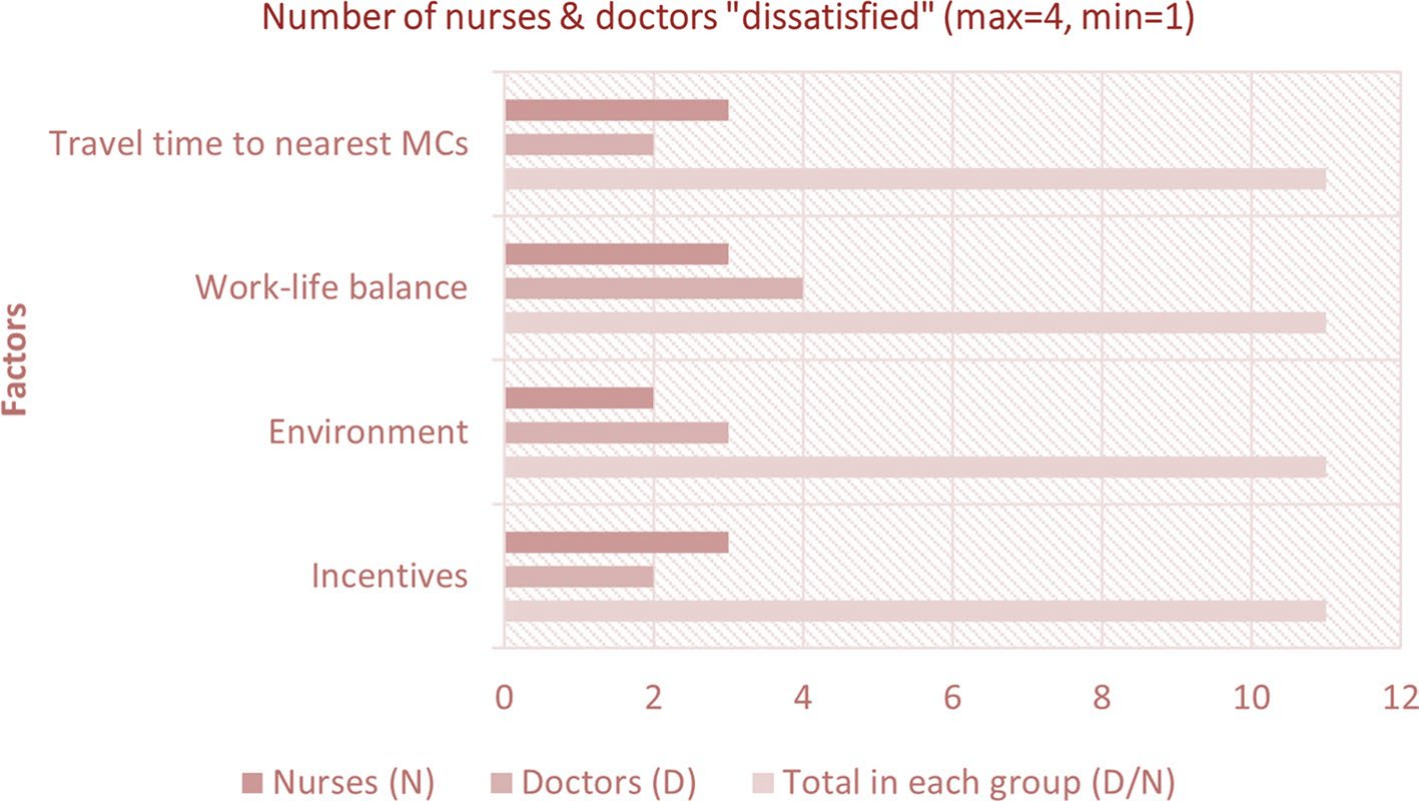
Horizontal bar graph illustrating the number of people (nurses and doctors) dissatisfied with the scheme and services of mohalla clinics considering four different factors

Figure 6 explicitly shows the number of nurses and doctors who were dissatisfied by the Mohalla Clinic health system. Four factors were found to be worth analyzing from the interviews which were incentives, environment, work-life balance, and travel time to nearest MCs. It is worth mentioning that 2 out of 11 (18 percent) doctors and 3 out of 11 (27 percent) nurses were dissatisfied by the incentives, 3 out of 11 (27 percent) doctors and 2 out of 11 (18 percent) nurses were dissatisfied by the environment, 4 out of 11 (36 percent) doctors and 3 out of 11 (27 percent) nurses were dissatisfied by the work-life balance, and 2 out of 11 (18 percent) doctors and 3 out of 11 (27 percent) nurses were dissatisfied by the travel time it takes to reach the designated Mohalla Clinic.

**Fig. 7:**
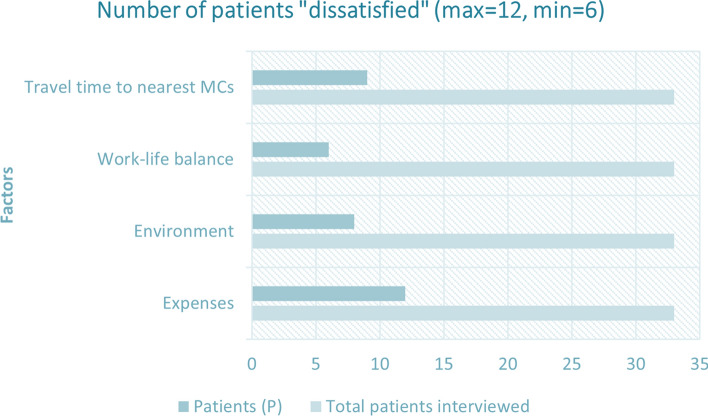
Horizontal bar graph illustrating the number of patients dissatisfied with the scheme and services of mohalla clinics considering four different factors

Figure 7 on the other hand shows the number of patients who were dissatisfied by the Mohalla Clinic health system. Four factors were found to be worth analyzing from the patients as well, which were incentives, environment, work-life balance, and travel time to nearest MCs. We found out that 12 out of 33 (36 percent) patients were dissatisfied by the expenses, 8 out of 33 (24 percent) patients were dissatisfied by the environment, 6 out of 33 (18 percent) patients were dissatisfied by the work-life balance, and 9 out of 33 (27 percent) patients were dissatisfied by the travel time it takes to reach the nearest Mohalla Clinic. Overall, we can conclude that although the percentage of dissatisfactions were very low in all user group interviewed (nurses, doctors, and patients) as compared to percentage of satisfaction, there is still scope of potential and improvement in the overall healthcare delivery system to achieve a desired level of satisfaction and thus achieving the gola of Universal health coverage (UHC). There were not many dislikes about the Mohalla Clinic healthcare system; thus, it can be said to be a state-of-the-art example that needs contextual modifications applicable in low-resource settings.

## Discussion

In Delhi, health care is provided through more than 25 different kinds of facilities such as apex or super specialty hospitals, specialty hospitals, a tertiary case including medical colleges hospitals, referral hospitals, district hospitals, sub-district hospitals, primary health centers, dispensaries, maternity homes, nursing homes, polyclinics, special clinics, chest clinics, venereal disease or sexually transmitted disease, mobile mother and child welfare units, mother and child welfare center, India population project clinics, postpartum departments, urban welfare centers, urban health posts, urban primary health centers, mobile medical team or mobile vans, maternity centers, school health clinics, and mobile dispensary. Even program managers have difficulty telling one facility from another regarding service delivery, and most of these buildings are virtually identical [9]. When viewed through the eyes of an outsider with inadequate knowledge, some people may mistake Mohalla Clinics for any other type of health facility. However, the Mohalla Clinics’ well-considered design distinguishes them from conventional healthcare facilities (Table 3).

It is imperative to address the harmonization of functions and convergence of multiple types of health facilities operated by different agencies because multiple health facilities make access to health services more difficult, time-consuming, and cumbersome for the public. Curative, clinical, or purely personal health services are the primary emphasis of most currently operating health facilities, leaving them underequipped to address the needs of the public or the general population.

## Role of Mohalla Clinics in the primary healthcare scenario

As part of this convergence, community people are more likely to accept Mohalla Clinics because they are in their neighborhoods and provide clinical services, which they see as desirable and needed services. Community members may react differently to public health services, and that’s where the Mahila Arogya Samiti (MAS) under National Urban Health Mission (NUHM) could be useful in this situation [32]. It’s expected that a well-functioning primary healthcare system will improve service delivery by lowering the cost of care. By providing primary healthcare for 80–90 percent of ailments, government health institutions (i.e., District hospitals) are freed up to provide expert treatments for needy people. Clinical and public health services must work together for the health sector’s efficiency. Doctors on “pay for service” contracts, rented premises for Mohalla Clinics, and flexible and varied clinic hours have all been implemented in Delhi to improve health service delivery. Also in the works is the utilization of medical college interns and postgraduate students for staffing chosen facilities, among other efforts. Information technology must be used to its fullest potential in these clinics and other healthcare facilities, as pharmaceutical vending machines are an example of this [33, 34]. The possibility to eliminate untrained caregivers, the decongestion of higher-level healthcare facilities, the availability of specialists for people who need them and the efficiency of healthcare delivery, as a result, are all aspects that these clinics have that are desirable in any health system. The approach applies to the entire country, not just Delhi, because these are problems that most Indian states face regarding their health systems. Patients can receive medical attention at these clinics because they are permanent healthcare institutions. People in the community will need to be counselled and transported to receive medical treatment soon if they receive preventive and promotion health services for emerging and re-emerging noncommunicable diseases and risk factors (e.g., diabetes, high blood pressure, various cancers, and ophthalmic issues). The government should not be discouraged from looking for ways to improve school health services by establishing these clinics, notwithstanding previous failures. Delhi’s schools house almost 40 million pupils [9], making it critical that Mohalla Clinics and educational institutions work together to improve the health of the city’s children.

An increasing focus on Universal Health Coverage (UHC) and primary health care is a vital component and accelerant for this movement. Community clinics’ expansion as a strategy for primary healthcare can help India reform health services and accelerate progress toward UHC, thus attaining the primary goal of the Indian National Health Policy [30]. Additionally, these hospitals must make public health, prevention, and promotion services available to provide comprehensive primary healthcare. The world’s experience shows that a single reform is insufficient and that a succession of linked reforms is always preferable. To make significant progress in healthcare reform, the federal government’s policy decisions and reform proposals must be implemented as soon as possible. Table 4 analyzes the UHC and health systems viewpoint by comparing population, quality of health services and financial protection.

**Table 4:** Mohalla clinics: analyzing using a universal healthcare and health systems viewpoint

Increasing population coverage	Increasing availability of quality health services	Financial protection and efficiency
1. Reduced travel and waiting time because of increased geographic access (opportunity cost). 2. Convenient office hours encourage people in the early stages of illness to seek care. 3. Enhanced access for unreachable and underserved populations including Jhuggi Jhopari (J.J.) clusters, resettlement colonies, and migrant basti’s. 4. Appropriate technology to meet regional medical requirements: token vending machines for patient queuing (equity) and electronic data maintenance for patient health records.	1. Providing a quality-assured package healthcare provided by trained professionals. 2. Give the people the option to choose a provider: potential to eliminate unqualified specialists. 3. Bring underserved individuals to mainstream health care system: possible change health seeking conduct. 4. Providing for the non-medical needs of individuals (response): the provision of drinking water and token vending reflects the machine and patient waiting area detailed design consideration	1. Reduced cost of care through the provision of guaranteed free medications and diagnostics: the cost of medicines and diagnostics accounts for nearly 70 percent of the healthcare expenditures of the general population. 2. Making services affordable for poor: accessibility reduces transportation costs and waiting time (the opportunity cost of missing work). 3. Interventions at a reasonable price: the price of services would be reasonable. By establishing an effective referral linkage in which 80 to 90 percent of health problems can be addressed at the community level. It has the potential to relieve pressure on higher-level health facilities.

## Limitations

Although the study provides the research community with an introduction of the Mohalla Clinics into the health landscape of Delhi with its history and detail of the scheme and how it unfolded and the potential it seems to offer, the study sample size of some Mohalla Clinics and types of users interviewed were relatively small due to several limitations. This is due to the fact of authors’ limits of asking users for voluntary participation to provide insights into their likes and dislikes with critics on how to improve the system. Author tried writing down all the answers in their raw form during the interview, but it might have impacted the interview flow and caused unforeseen data loss. The research setting, the Mohalla Clinics, are always a busy point in the street (where people wait from early morning in line, waiting for the Mohalla Clinic to open at its scheduled time), making the circumstances unwelcoming for research enquiry.

## Conclusion

The study attempts to investigate Delhi’s healthcare system through its Mohalla Clinics initiative of providing key healthcare services to remote populations. The case study clearly states the strengths and limitations of the Mohalla Clinic system’s lack of the good goal of Universal Health Coverage (UHC).

If we closely analyze the whole operationalization of Mohalla Clinics with several advantages as already discussed benefits and working in conjunction with the dispensaries, UPHC, and polyclinics only for urban India. Following are some recommendations for the primary healthcare sector to achieve Universal Health Coverage (UHC).

- Create a comprehensive road map and operational plan: including technical, financial, and administrative considerations. The government may contemplate making this accessible to the public with measurable indicators and timelines.
- Establish performance monitoring and evaluation mechanisms: utilize data from these facilities for analysis in real-time
- Avoid duplication even if it is politically appealing: utilize the current dispensaries as Mohalla Clinics or polyclinics. The operation of these dispensaries should be analyzed, and then they should be assigned an appropriate role within the system of health service delivery in Delhi. Whereas Mohalla Clinics concentrate on clinical services, other National Urban Health Mission (NUHM) facilities, such as Urban Primary Health Centres (UPHC), provide complementary public health services.
- Guarantee political buy-in and financial viability: engage with key stakeholders, such as political parties, community leaders, councilors, and other stakeholders. Construct or at least attempt to construct consensus.

**Fig. 8:**
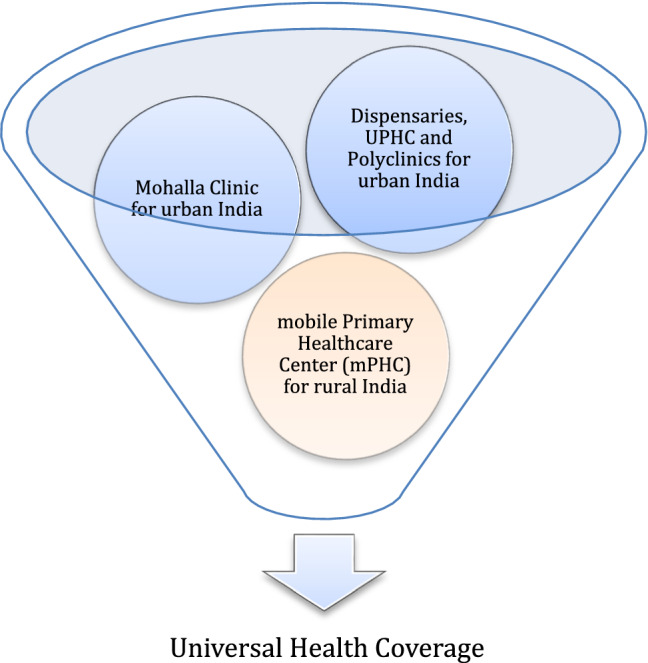
A conceptual model showing the proposed mobile Primary Health Center (mPHC) as the missing component to complete the Universal Health Coverage (UHC)

Universal Health Coverage (UHC) calls for healthcare access for all at affordable cost. Rural India still has not been served with the principles of UHC and we found a missing element apart from the dispensaries, polyclinics, UPHCs and Mohalla Clinics. Primary healthcare in the Indian context is only limited to urban regions. The remote population is still underserved due to several limitations, such as finances, poor road connectivity, etc. Thus, the proposed idea of making the Primary Health Center (mPHC), a mobile infrastructure, a collapsible system, will act as the missing element to complete the gap in Universal Health Coverage (Fig. 8).

“*Healthcare on Wheels—Mobilizing healthcare to the doorstep of the remote populations—A lesson from the Covid-19 pandemic. The Indian healthcare system urgently requires a new healthcare delivery model to improve healthcare accessibility and health outcomes for the marginalized. People living in remote areas are disproportionately affected by the inaccessibility and underutilization of Primary Health Centers (PHCs). The goal is to design, develop and deploy a cost-effective collapsible mobile Primary Health Center (mPHC) unit in low resource settings. A traditional Primary Health Center (PHC) activities will be decentralized into several modules for ease of deployment and reachability to remote locations.*”—Author

The proposed concept of a collapsible system for a mobile Primary Health Center (mPHC) is intended to be carried in bags to remote regions, deployed in less than 60 min, run OPD. for 4–6 h, collapse, and return to the base camp. It is based on patient-centered care, delivering healthcare to the underserved doorstep. Most of the system’s elements must be collapsible by design for it to be collapsible. As a result, it is critical to focus on the design of sub-systems to be as collapsible as possible to take up the least amount of space for easy transport. These conclusions were drawn from the analysis and examination of Mohalla Clinics’ strengths and limitations, as well as a recommendation of future measures in a vision to promote India’s aim of Universal Health Coverage (UHC). It is expected that the findings would pave ways to develop and implement such facilities in low resource settings.

## Data Availability

All data generated or analyzed during this study are included in this published article. Qualitative interviews were reported as a summary so that the article could also incorporate other dimensions to the study from literature such as the Universal Health Coverage.

## References

[1] Menabde N, Lahariya C (2015). India’s draft national health policy, 2015: improving policy to implementation effectiveness. J Fam Med Prim Care.

[2] Lahariya C (2017). Mohalla clinics of Delhi, India: could these become platform to strengthen primary healthcare?. J Fam Med Prim Care.

[3] AAP government will set up 1000 mohalla clinics by March 2017: Arvind Kejriwal. 2016. https://indianexpress.com/article/india/aap-government-will-set-up-1000-mohalla-clinics-by-march-2017-cm-arvind-kejriwal-4406863/. Accessed 25 Nov 2022.

[4] Delhi citizens handbook. 2016. https://ccs.in/download.php?file=sites/all/books/com_books/dch-2016.pdf. Accessed 25 Nov 2022.

[5] Prakash R. Karnataka to replicate Delhi’s mohalla clinics. Times of India. 2016. https://timesofindia.indiatimes.com/city/bengaluru/karnataka-to-replicate-delhis-mohalla-clinics/articleshow/55095782.cms. Accessed 25 Nov 2022.

[6] Sharma R. Eye on polls, Gujarat govt to set up ‘mohalla clinics’ in 4 cities, Indian Express. 2016. https://indianexpress.com/article/cities/ahmedabad/eye-on-polls-gujarat-govt-to-set-up-mohalla-clinics-in-4-cities-3069359/. Accessed 25 Nov 2022.

[7] Lahariya C (2016). Delhi’s mohalla clinics: maximising potential. Econ Polit Wkly.

[8] Gusmano MK, Rodwin VG, Weisz D (2017). Delhi’s health system exceptionalism: inadequate progress for a global capital city. Public Health.

[9] Territory of Delhi statistical abstract of Delhi 2022. http://des.delhigovt.nic.in/wps/wcm/connect/9e42f900409730e68a0abea50c073453/SAbstract2022.pdf?MOD=AJPERES&lmod=1024529098&CACHEID=9e42f900409730e68a0abea50c073453&StatisticalAbstarct2022. Accessed 25 Nov 2022.

[10] Statement No 16.1 health infrastructure facilities in Delhi during the period 2010–2017. Econ. Surv. Delhi. 2010, p. 2018–2037. http://delhiplanning.nic.in/sites/default/files/ES%202017-18%20Health.pdf. Accessed 25 Nov 2022.

[11] Gururaj G, Varghese M, Benegal V, Rao GN, Pathak K, Singh LK. National health profile (NHP) of India—2019: Ministry of Health and Family Welfare. 2016. http://cbhidghs.nic.in/showfile.php?lid=1147. Accessed 25 Nov 2022.

[12] Government of India. Key indicators of social consumption in India Health NSS 71st round. Natl. Sample Surv. Organ. 2015, p. 1–99. http://mospi.nic.in/sites/default/files/publication_reports/nss_71st_ki_health_30june15.pdf; http://mail.mospi.gov.in/index.php/catalog/161/download/1949. Accessed 25 Nov 2022.

[13] Sharma DC (2016). Delhi looks to expand community clinic initiative. Lancet.

[14] Dutt A. Seven things to know about Delhi’s mohalla clinics praised by world leaders. Hindustan Times. 2017, p. 6–10. http://www.hindustantimes.com/delhi/7-reasons-why-world-leaders-are-talking-about-delhi-s-mohalla-clinics/story-sw4lUjQQ2rj2ZA6ISCUbtM.html. Accessed 25 Nov 2022.

[15] Kofi Annan lauds AAP mohalla clinics project, suggests reforms. 2017. https://www.hindustantimes.com/delhi/kofi-annan-lauds-aap-s-mohalla-clinics-project-suggests-reforms/story-UMTbFhQ1KY9tyaExxHS3kK.html. Accessed 25 Nov 2022.

[16] Ministry of Health and Family Welfare. Brief write up on Aam Aadmi Mohalla Clinic. 2020. http://health.delhigovt.nic.in/wps/wcm/connect/doit_health/Health/Home/Directorate+General+of+Health+Services/Aam+Aadmi+Mohalla+Clinics. Accessed 25 Nov 2022.

[17] Rao M. The clinic at your doorstep: how the Delhi government is rethinking primary healthcare. Scroll.in. 2016. https://scroll.in/pulse/807886/the-clinic-at-your-doorstep-how-the-delhi-government-is-rethinking-primary-healthcare. Accessed 25 Nov 2022.

[18] Mabuto T, Latka MH, Kuwane B, Churchyard GJ, Charalambous S, Hoffmann CJ (2014). Four models of HIV counseling and testing: utilization and test results in South Africa. PLoS ONE.

[19] Labiris G (2003). Tele-ophthalmology and conventional ophthalmology using a mobile medical unit in remote Greece. J Telemed Telecare.

[20] Van Dijk JH, Moss WJ, Hamangaba F, Munsanje B, Sutcliffe CG (2014). Scaling-up access to antiretroviral therapy for children: a cohort study evaluating care and treatment at mobile and hospital-affiliated HIV clinics in rural Zambia. PLoS ONE.

[21] Abolfotouh MA, Al-Assiri MH, Al-Omani M, Al Johar A, Al Hakbani A, Alaskar AS (2014). Public awareness of blood donation in central Saudi Arabia. Int J Gen Med.

[22] P. BS. Cabinet approves Delhi healthcare corporation. The Hindu, Delhi. 2015. https://www.thehindu.com/news/cities/Delhi/cabinet-approves-delhi-healthcare-corporation/article7709317.ece. Accessed 25 Nov 2022.

[23] L. Updated. AAP-centre tussle ruled in 2016, ball in SC’s court in 2017. 2016. https://www.business-standard.com/article/news-ians/aap-centre-tussle-ruled-in-2016-ball-in-sc-s-court-in-2017-2016-in-retrospect-116121900264_1.html. Accessed 25 Nov 2022.

[24] Yu SWY, Hill C, Ricks ML, Bennet J, Oriol NE (2017). The scope and impact of mobile health clinics in the United States: a literature review. Int J Equity Health.

[25] Times H. Eight lakh treated in five months at Delhi mohalla clinics. New Delhi, 2016. https://www.hindustantimes.com/delhi/eight-lakh-treated-in-five-months-at-delhi-mohalla-clinics/story-KQl2baCrJ5lz8TS3ZmuziN.html. Accessed 25 Nov 2022.

[26] Indian Express. Delhi: dengue testing facility at mohalla clinics from next month. New Delhi Indian Express. 2016. https://indianexpress.com/article/india/india-news-india/delhi-dengue-testing-facility-at-mohalla-clinics-from-next-month-2966750/. Accessed 25 Nov 2022.

[27] The Lancet (2016). Universal health coverage looking to the future. Lancet.

[28] Wadhwa. What New Delhi’s free clinics can teach America about fixing its broken health care system? Washington Post. 2016. https://www.washingtonpost.com/news/innovations/wp/2016/03/11/what-new-delhis-free-clinics-can-teach-america-about-fixing-its-broken-health-care-system/. Accessed 25 Nov 2022.

[29] AAP to inaugurate its first mohalla clinic in city, 2016. https://www.dnaindia.com/mumbai/report-aap-to-inaugurate-its-first-mohalla-clinic-in-city-2247324. Accessed 25 Nov 2022.

[30] Lahariya C (2016). Abolishing user fee and private wards in public hospitals. Econ Polit Wkly.

[31] Chan P, San S. Towing AAP line, PMC mulls mohalla clinics. 2016. https://punemirror.com/pune/civic/towing-aap-line-pmc-mulls-mohalla-clinics/cid5236426.htm. Accessed 25 Nov 2022.

[32] Ministry of Health and Family Welfare (Government of India). National urban health mission: framework for implementation. 2013, p. 80. http://nhm.gov.in/images/pdf/NUHM/Implementation_Framework_NUHM.pdf. Accessed 25 Nov 2022.

[33] Delhi: Mohalla clinic gets a medicine dispensing machine. 2016. https://www.hindustantimes.com/delhi/delhi-mohalla-clinic-gets-a-medicine-dispensing-machine/story-Hui5HPRP4cKI0rehE4oujM.html. Accessed 25 Nov 2022.

[34] U.S. Embassy. First-of-its-kind medicine vending machine inaugurated at Aam Aadmi Mohalla Clinic. 2016. https://in.usembassy.gov/first-kind-medicine-vending-machine-inaug-aam-aadmi-mohalla-clinic-jt-initiative-delhi-govt-usaid-wish-q/. Accessed 25 Nov 2022.

